# Involving Independent Mental Capacity Advocates (IMCAs) in Dental Care Decision‐Making: A Qualitative Exploration of Dentists’ Experiences in England and Wales

**DOI:** 10.1111/scd.70276

**Published:** 2026-10-22

**Authors:** Naomi Murray, Jeena Patel, Lucy Taylor, Jordan A. Parsons, Andrew Geddis‐Regan

**Affiliations:** ^1^ University Dental Hospital of Manchester Manchester University NHS Foundation Trust Manchester UK; ^2^ Department of Special Care Dentistry Barts Health NHS Trust London UK; ^3^ Department of Special Care Dentistry Oxford Health NHS Foundation Trust Oxford UK; ^4^ St. Mary's University School of Medicine London UK; ^5^ Division of Dentistry University of Manchester Manchester UK

**Keywords:** clinical ethics, decision‐making, dental care, health services research, patient care planning, patient participation, special care dentistry, treatment planning

## Abstract

**Introduction:**

Delivering dental care to individuals who lack decision‐making capacity involves complex ethical, legal, and clinical considerations. In England and Wales, Independent Mental Capacity Advocates (IMCAs) are required to support decisions about ‘serious medical treatment’, a concept given only broad definition in law. While Special Care Dentistry frequently involves patients with cognitive impairments, little is known about how dentists engage with advocates in treatment decision‐making.

**Objective:**

To explore dentists’ experiences of appointing and working with advocates in dental care decision‐making and to understand the reasons for advocacy involvement and its perceived value.

**Methods:**

Semi‐structured one‐to‐one online interviews were conducted with nine dentists. Data were generated and analyzed concurrently using a maximum variation sampling approach and Reflexive Thematic Analysis. Interviews were audio‐recorded, transcribed verbatim, and initially coded independently by four researchers. Through iterative discussion, codes were refined and organized into themes.

**Results:**

Five themes were generated. Dentists demonstrated a strong commitment to optimizing patient capacity and delivering person‐centered care. However, there was substantial variation regarding the interpretation of ‘serious medical treatment’ in the dental context. Decisions to involve IMCAs were often based on treatment modality rather than individual patient impact. Dentists reported that advocates’ involvement rarely altered proposed treatment plans and felt this was frequently associated with delays, which were perceived as counterproductive to patient care.

**Discussion:**

Uncertainty surrounding the definition of ‘serious medical treatment’ contributes to inconsistent advocate involvement and may limit the effectiveness of advocacy. A broader, individualized understanding of seriousness, incorporating clinical, functional, and personal consequences, is needed.

**Conclusion:**

Clearer guidance and earlier, more meaningful advocate involvement are needed to support consistent, timely, and genuinely person‐centered dental decision‐making for patients without friends or family to support them.

## 1 Introduction

Special Care Dentistry (SCD) addresses the oral health needs of vulnerable groups, many of whom have cognitive impairments requiring complex or adapted care [1]. Patients with a range of conditions that can impair decision‐making capacity are supported by SCD teams, including learning disabilities, dementia, and mental illness [2]. These conditions are associated with poorer oral health outcomes, resulting in a disproportionate burden of dental disease among individuals who may lack the capacity to consent to treatment [3, 4, 5].

Research into treatment decision‐making in SCD is a limited but growing area of study [6, 7, 8]. Dental interventions offer benefits including pain relief and restored function, but can also carry significant risks. For example, tooth extraction can impact appearance, identity, and psychosocial wellbeing, requiring careful balancing of benefits and harms in light of each individual's values [7, 9].

In England and Wales, the Mental Capacity Act 2005 (MCA) requires decisions on behalf of adults who lack capacity to be made in the person's best interests [10]. In determining best interests, the decision‐maker must, where practicable, consult those close to the individual, such as family members or unpaid carers, about the person's wishes, feelings, beliefs and values [10, 11]. Crucially, individuals engaged in providing care or treatment in a professional capacity or for payment cannot be the sole individuals to fulfil this consultative role, however well they may know the person.

Where a public health body in England or Wales proposes “serious medical treatment” for a person who lacks capacity and has no family or friends whom it is appropriate to consult (a situation sometimes described as being “unbefriended”), it has a statutory duty to instruct an Independent Mental Capacity Advocate (IMCA) before the decision is made [10, 11, 12]. Serious medical treatment is defined in regulations as treatment which involves providing, withdrawing or withholding treatment where there is a fine balance between its benefits and its burdens and risks, where a choice between treatments is finely balanced, or where what is proposed would be likely to involve serious consequences for the patient [12].

IMCA services are commissioned locally and delivered by independent advocacy organizations, from which an advocate is allocated on referral, typically meeting the person, examining relevant records, consulting others involved in their care, and producing a report the decision‐maker must consider. Importantly, the IMCA is an advocate, not a decision‐maker: they articulate the person's wishes, feelings, beliefs and values and may challenge the proposed course, but they neither make the best interests decision nor authorize treatment, which remains the responsibility of the treating clinician [10].

The approach in England and Wales contrasts with Scotland's clinician‐only model [13], and most other jurisdictions' substitute decision‐maker frameworks [14, 15]. The United States lacks a uniform approach; instead, states employ diverse mechanisms for unbefriended patients, from unilateral physician authority to guardianship systems [15]. The IMCA model is distinctive in inserting independent advocacy into a process where the clinician remains the decision‐maker.

Dental treatment can be delivered through a range of modalities [16], such as local anesthesia, conscious sedation, or general anesthesia (GA). Dentists vary substantially in their IMCA appointment thresholds, and this inconsistency across professional networks remains poorly understood. This study explores the reasons behind this variation and identifies how consistent, high‐quality care can be facilitated for adults with cognitive impairment.

## 2 Aim

This study aimed to explore dentists’ experiences of appointing and working with IMCAs in dental treatment decision‐making, including perceptions of the IMCA's role, the circumstances prompting appointment, and the value and limitations of IMCA input.

## 3 Methods

### 3.1 Design and Approach

A qualitative research approach was used to explore dentists’ experiences and perceptions of IMCA involvement in dental decision‐making, grounded in a constructivist epistemology in which meaning is understood as co‐constructed through social interaction and researcher interpretation [17]. Semi‐structured one‐to‐one interviews were chosen as they allow for participants to be prompted through areas of interest whilst allowing them to explain their perspectives freely.

This study received ethical exemption from the University of Manchester, as it involved qualified professionals participating in the context of their professional roles (reference: 2025‐23392‐42623).

### 3.2 Recruitment and Sampling

Potential participants were identified through direct approach, local professional networks, and the national specialty trainee network for SCD, subject to specific inclusion and exclusion criteria: dentists had to be qualified and registered with the UK General Dental Council, working in England and Wales where the MCA applies, with experience assessing, treating, referring, or managing patients with impaired capacity. Dentists in Scotland or Northern Ireland were excluded, as the MCA does not apply there. Eligible dentists were sent a participant information sheet and invited to express interest.

Maximum variation purposive sampling was used, operationalized across four dimensions relevant to IMCA involvement: clinical role (dental officers, senior dental officers, specialty trainees, specialists, and consultants), service setting (community and hospital dental services^1^), geographical region across England and Wales, and years since qualification. Thirteen dentists were approached, of whom nine participants were interviewed. The sample size was not predetermined; data generation continued until the research team reached agreement that sufficient information power was attained [18], in that the analytical understanding of the topic was sufficiently developed and that further interviews were unlikely to enhance this. As each interview was completed and transcribed, the team reviewed the initial generated data prior to subsequent interviews: by the later interviews, coding was generating no new patterns, and subsequent transcripts were elaborating and nuancing the developing analysis rather than disrupting or extending it. The specificity of the sample's dense, directly relevant experience of IMCA involvement, and the quality of interview dialogue meant the team judged sufficient information power to have been attained. Given recruitment through professional networks, prior acquaintance between interviewers and some participants was possible. Interviewers were made aware of this dynamic and encouraged to reflexively consider assumptions arising from existing professional relationships in analysis processes.

### 3.3 Data Generation

The research team was led by a consultant in special care dentistry (AGR) and an academic health law specialist (JAP), both with extensive qualitative research experience, together with three specialty registrars in SCD (NM, JP, LT) who were new to qualitative research; all clinicians had experience of appointing and working with IMCAs. Interviews were conducted by NM, JP and LT (all female) via Microsoft Teams one‐to‐one, with no third parties present and informed consent obtained beforehand. Participants knew the interviewers were dentists themselves. A topic guide was developed and iteratively refined through piloting with clinical colleagues not involved in the study (please see Supplementary File 1 for additional information) Interview conduct was supported by the two senior researchers, with transcripts reviewed as data generation progressed so the approach could be refined across the series. Interviews were audio‐recorded, transcribed verbatim, then recordings were deleted. Transcripts were pseudonymized for analysis.

### 3.4 Data Analysis and Reflexivity

Data were analysed using inductive reflexive thematic analysis, within which researcher subjectivity is understood as a resource for interpretation rather than a source of bias to be controlled [19, 20]. Four authors (AGR, NM, JP, LT) independently applied initial open codes to each transcript, intended not to establish agreement but to allow researchers with differing positionalities (special care dentists in different services, and an academic health law specialist without a clinical background) to read the data through distinct interpretive lenses before these were brought into iterative collective dialogue.

Authors used either NVivo 15 (QSR International, 2020) or Microsoft Excel (Microsoft Corporation, 2018) to organize coded data. Points of divergence between researchers’ coding were treated as analytically generative and explored in collaborative team meetings, first between AGR and NM and subsequently involving AGR, LT and JP, in which assumptions underpinning codes were questioned, provisional categories examined, and candidate themes actively constructed through reflexive dialogue.

Themes were further reviewed and refined during write‐up by JAP and AGR, with writing itself treated as a continuation of the analysis. Transcripts were not returned to participants and member‐checking was not undertaken; given our epistemological position that themes are constructed through interpretation rather than extracted from data, either practice would have been inconsistent with the approach adopted. Data generation and analysis proceeded simultaneously, consistent with the principles of reflexive thematic analysis [20].

## 4 Results

Nine interviews were completed over three months (November 2025 to January 2026). Participants were dentists from across England and Wales, working in a range of roles within special care dentistry, from community dental officers to consultants, qualifying between 8 and 26 years previously. Interview duration ranged from 25 to 81 min (mean 44 min). Participant characteristics are summarized in Table 1. Five themes were generated from the data.

**TABLE 1:** Demographics of dentists interviewed.

Participant No.	Gender	Years qualified	Job role	Practice setting	Location
1	F	20 to < 30	Senior dental officer	Community dental services	Midlands, Wales
2	F	20 to < 30	Specialist in SCD	Community dental services	Southern England
3	F	10 to < 20	Consultant in SCD	Hospital	London
4	F	20 to < 30	Consultant in SCD	Hospital	London
5	M	5 to < 10	Dental officer	Community dental services	Midlands
6	F	5 to < 10	Specialist trainee in SCD	Hospital	Northern England
7	F	20 to < 30	Specialist in SCD	Community dental services	Southern England
8	M	20 to < 30	Specialist in SCD	Community dental services, Hospital	Southern England
9	M	5 to < 10	Specialist in SCD	Community dental services, Hospital	Wales

*Note*: Demographic detail has been limited to protect anonymity.

### 4.1 Supporting and Optimizing Capacity

Capacity assessment was described as a routine, embedded component of SCD practice. Dentists demonstrated clear familiarity with their legal obligations to presume capacity and support it before concluding otherwise, approaching this not as a burden but as a core clinical skill. Personalized, adapted communication strategies were central to this:

“my first port of call would just be trying to encourage any capacity that they do have. So, putting things in really simple terms, even just doing kind of the social stories, with books and things like that. So I can, I can at least say that I've put every kind of adjustment I can in place to encourage that from them just so that they understand what's involved, but even you know, with the best will in the world, that might not be possible with some patients.” (P5)

Creative adjustments sometimes yielded insights that might otherwise have been missed:

“There have been cases where I would use like picture cards. So, I remember this one case of the gentleman's cerebral palsy. He was giving kind of three out of four times. He would say something consistently, but was that one of the four? So, I was like, let's use picture cards and he's able to point to the same thing consistently that way, which suggests to me that he did have capacity.” (P9)

Where an individual was deemed to lack the requisite capacity, IMCA appointment was described as a last resort following thorough assessment. Uncertainty about capacity prompted collaborative review, with second opinions and reassessment regularly employed:

“If I'm really stuck and wasn't sure whether this patient had the capacity, I'd probably get a second opinion of a colleague just to confirm my thinking…I know it's very decision specific, but if I was in doubt, I would get the advice of a colleague and look at the broader history of the patient.” (P4)

Participants consistently described capacity support as preceding consideration of IMCA involvement. Reasonable adjustments and adapted communication were seen as essential because successful support could enable a person to decide for themselves, removing the need for best interests processes and independent advocacy altogether. IMCA appointment was accordingly framed as a safeguard reserved for situations where, despite these efforts, the person was assessed as lacking decision‐specific capacity and the statutory criteria for advocacy were met.

### 4.2 Inconsistent Interpretation of Advocacy Requirements

What constitutes ‘serious medical treatment’ in the dental context proved highly contested. Dentists described navigating this ambiguity without clear guidance, with the form of dental behavior support [21] employed becoming the dominant proxy:

“So an IMCA is required for serious medical treatment and unbefriended people. The reason it's not clear is what is serious medical treatment? Most people don't know what that is. I still don't know what that is.” (P9)

GA and intravenous sedation were most consistently identified as the threshold, while local anesthesia alone was generally not considered serious treatment:

“I'm doing a GA, I'm getting an IMCA involved. If I'm doing sedation, I'm getting an IMCA involved. Maybe if it's just gas and air and they're really medically fit and well and they've had it fifteen times before maybe not, but certainly IV sedation or GA anything, whether there's a risk to life and well‐being then, yeah, of course, no question that's serious.” (P8)

Even within this consensus, reported practice varied:

“I would get an IMCA for any GA procedure for a patient who lacks capacity and is unbefriended…for the first year and a half there, we wouldn't even go that far; we would only get an IMCA if there was a dispute between the care team and what we propose, or if the risks were unclear.” (P9)

The functional and personal consequences of treatment carried less consistent weight:

“Unless you were going to take out every single tooth in a patient's mouth and that could affect their eating, for example. But I still wouldn't consider that as serious medical treatment.” (P4)

Where treatment could be delivered under local anesthesia, participants described proceeding with treatment without IMCA appointment, typically regardless of the extent or irreversibility of the treatment itself. Treatment modality therefore functioned not merely as a proxy for seriousness but, for many participants, as its practical definition: dental treatment delivered without sedation or GA appeared not to be conceived of as “serious medical treatment” at all, however significant its functional or personal consequences. This reflects a broader pattern in beliefs surrounding GA in special care dentistry, whereby GA itself, rather than the treatment it enables, is often treated as the locus of risk and seriousness requiring justification [6, 7].

A minority of dentists articulated an explicitly individualized understanding of seriousness, one in which the impact of a treatment decision could only meaningfully be assessed relative to that person's own life, identity, and circumstances. This perspective, illustrated in detail by P8, most closely reflects the spirit of person‐centered best interests assessment as envisaged by the MCA:

“[seriousness] it's down to the individual. Let's conjecture somebody who's got 28 teeth and they're 18 years old, in residential care since mum died, and they've fractured their upper incisor. You're talking about taking that out… Now let's talk about person B. He's 65, he's got autism and profound learning disability, he never looks in the mirror and has no interest in his own self‐image. I don't think taking out that extremely loose upper incisor is going to change his quality of life one iota. It feels a bit more individual, that decision.” (P8)

The quotation does not suggest that IMCA involvement would automatically follow for the younger patient. Rather, it illustrates how participants considered the individual's circumstances and the significance of the consequences when assessing whether a decision should be regarded as sufficiently serious to warrant the additional input of an IMCA. The emphasis was on the personalized assessment of seriousness, which could influence both decision‐making and consideration of statutory processes, rather than on the dental procedure itself.

In emergencies, IMCA appointment was set aside:

“Obviously emergencies are different… it's, you know, emergency surgery for example, then you know I think the IMCA scenario becomes irrelevant.” (P2)

General anesthesia is rarely required urgently in SCD; urgent care here means emergency or unplanned access to theatre, which is typically provided by hospital‐based maxillofaical surgery teams in the UK context. Most SCD GA treatment is delivered electively, though patients with significant pain or infection are prioritized for the earliest available lists within service capacity. Consistent with this, no participant described currently bypassing IMCA involvement where GA was planned, as time was typically available to undertake advocacy processes.

### 4.3 Advocacy of Uncertain Value and Depth

Experiences of working with IMCAs varied considerably. Some described positive, proactive advocacy that enriched the process:

“[The IMCA] asked some really fantastic questions and I feel really advocated for the patient in ensuring that all the options were on the table and the risks and benefits of each one were discussed.” (P1)

Others perceived IMCAs to lack understanding of dental care, with a cultural undervaluing of dentistry appearing to influence engagement:

“If it's one tooth that might not be deemed as serious to them. I think maybe in dentistry specifically, there's always that kind of ethos ‘oh it's just teeth’. You know, it's not like it's heart surgery, brain surgery, that kind of thing. So I think it can be a little bit ambiguous in that way.” (P5)

In some cases, IMCA actions led to confusion and a lack of progress:

“I have heard of situations where IMCAs have maybe overstepped, such as one case where an IMCA found a video about local anesthetics to show a patient, which completely unsettled them and undid quite a bit of good work done in the clinic.” (P2)

Physical and relational distance between dental teams and IMCAs was a recurring feature, limiting shared understanding:

“Previously they definitely met them at their home, but they were also patients that were known to the IMCAs because they had been involved in decisions around accommodation and hospital stays. On no occasion have I ever met with them in a clinic. They have been part of virtual best interest discussions, but I have never met them in person.” (P7)

Variability in IMCA engagement mirrored variability in appointment decisions, reflecting a systemic absence of shared expectations for advocacy in this specialist context. Participants suggested several explanations: IMCAs’ limited familiarity with dental treatment and terminology, which made substantive engagement difficult; a physical and relational distance from the treating team, with contact sometimes limited to brief conversation or written correspondence; and the absence of any shared professional expectation for what dental advocacy should look like. Underlying several accounts was a sense that this variability reflected the broader marginalization of oral health within decision‐making, echoing the view of dental treatment as inherently less serious discussed elsewhere in these findings.

### 4.4 Pre‐Determination of Plan

IMCA involvement rarely altered the dentist's pre‐existing plan.

“[The treatment plan] is obvious. I don't see what an independent non‐healthcare kind of person would be able to add to that unanimously agreed plan.” (P9)

“Clinicians like myself still have a huge amount of power which, you know, fills me with no great excitement…it weighs heavily because I'm always conscious as the decision maker as defined by the MCA, you know you draw on your experience and training.” (P8)

These accounts illustrate contrasting relationships to the power the MCA vests in the clinician. P9 frames the plan as already settled by clinical consensus, positioning the IMCA as external to a decision effectively already made; the “unanimity” invoked was reached among clinicians, not with the patient. P8, by contrast, names himself explicitly as decision‐maker under the MCA and describes that authority as held uneasily rather than confidently, suggesting awareness of the power imbalance the IMCA exists to offset. Where a contribution is understood as additive to an already‐complete judgement, as in P9's account, its capacity to influence the plan is constrained before the IMCA is even instructed; where it is understood as a check on the clinician's own authority, as in P8's account, engagement might look different. This distinction may help explain the variation in perceived value described elsewhere.

Participants described proceeding with care planning in parallel with the IMCA process:

“I would put a patient on a waiting list, assuming we'd get the go‐ahead, so that their wait isn't impacted, rather than waiting for their response from the IMCA — and in my experience it has supported our best interests decision.” (P9)

In one instance, an IMCA's persistence materially changed the plan, producing a more person‐centered outcome:

“[The IMCA] challenged the consultants in a very professional way but didn't accept you know the first ‘no I don't think that's a good option’ because this person needs lots of treatment… there was a toing and froing… this IMCA kept saying ‘but if they have the support in place and they can come to the appointments as difficult and challenging as they may be, why can we not give the intravenous sedation’ and it just, yeah. And I think it took some time, but that was the agreement in the end and I was quite impressed because I don't think I would have challenged it quite as long as she did.” (P1)

The dominant picture described was one of IMCA involvement confirming rather than challenging existing plans.

### 4.5 Paradoxical Delays to Care

Care delays due to IMCA appointment were reported by every participant:

“Yeah, it's delayed everything and it's added more workloads to us and it hasn't changed the outcome of the patient or it's hindered the outcome of the patient.” (P4)

Where the IMCA process ran in parallel with existing waiting times for GA, which are often substantial in publicly funded dental services, the additional delay was reported as less problematic. Some services had adapted their processes accordingly:

“If you've got quick decisions that need to be made. If, and this is the case for our service, most of the IMCA involvement is around decision making for general anesthetic, there is a bit of leeway of time that we have before a patient will get to the top of the waiting list anyway, so I guess it depends on how long the current provider takes to come up with a report”. (P7)

This pragmatic adaptation reflected dentists’ recognition that IMCA involvement rarely altered a clinical decision. Crucially, these delays fall on a group least able to absorb them. Adults who lack capacity and are unbefriended often already experience compounded barriers to timely dental care, including communication difficulties, reliance on others to recognize need, and limited routine access [22]. A safeguard intended to protect their interests can therefore paradoxically deepen the very disadvantage it exists to counter, prolonging pain, infection, or deterioration while the process runs its course. Rather than functioning purely as a protection, advocacy in this context can become an additional hurdle, imposing the heaviest procedural burden on those with the greatest need and the least resilience.

## 5 Discussion

This study offers the first in‐depth exploration of dentists’ experiences involving advocates in dental decision‐making, highlighting a field characterized by legal obligation, clinical commitment, and systemic ambiguity.

A central finding is the consistent pre‐determination of dental care plans prior to, and largely unaffected by, IMCA involvement, although dentists did typically value IMCAs’ role and input; this contrasts with findings from inpatient hospital settings [23]. There are nonetheless parallels with other healthcare research, where clinicians’ approaches to decision‐making for people with cognitive impairment were similarly shaped by professional judgement and clinical confidence [7, 24]. While this may reflect genuine expertise in adapting care to individual needs, it also raises concern that treatment misaligned with a person's preferences may be facilitated [8] and that independent advocacy's core aim, amplifying the individual's voice, is not being fully realized.

The variable value participants placed on IMCA involvement cannot be attributed to a single cause: structural factors, such as physical distance between dental teams and advocates, and practice‐level factors, such as inconsistent engagement, both featured in accounts. However, the recurring framing of dental treatment as inherently minor, “just teeth” in one participant's words, suggests that clinician perception of the specialty itself may constrain how much value is attributed to advocacy from the outset, independent of any individual advocate's conduct. This aligns with the pre‐determination of plans discussed above, and raises whether advocacy is undervalued because it functions poorly here, or because dentistry itself is not viewed as a domain where independent representation is expected to matter.

Understanding how profoundly dental conditions or care sequelae impact a person is highly individual. For people with static cognitive impairments, such as severe learning disabilities, personal factors may not be simple to ascertain. When a person has a dynamic cognitive impairment and previously had no such impairment, any known preferences and biographical context can aid value‐informed decision‐making [25]. Dental teams and advocates alike have a duty to seek out and center this information rather than defaulting to a treatment plan constructed purely on clinical grounds [26, 27].

The ambiguity surrounding ‘serious medical treatment’ (SMT) in the specific dental context is a significant and unresolved problem. The MCA Code of Practice [11] and IMCA regulations [12] provide only broad parameters. Whilst some participants spoke to the law's focus on finely balanced decisions, case law provides little by way of clarifying the dental determinants of seriousness in relation to consequences of care. This challenge can be exacerbated when the consequences of care may not be known, specifically for those who may be intolerant of clinical and radiographic examination without pharmacological support. In this study, GA was the most common trigger for IMCA appointment, but this modality‐based heuristic is an imperfect proxy: the MCA intends SMT to encompass the functional, psychological, and social consequences of both treatment and non‐treatment for that specific person [28, 29].

This mirrors the equally contested nature of ‘best interests’ under the MCA [24, 28]. Best interests determination requires synthesis of the person's wishes, values, and biographical context alongside clinical evidence. The risk of conflating legal with clinical best interests is well documented, including in evidence to the House of Lords Select Committee on the MCA [30], and was addressed by the Supreme Court in *Aintree* [31], which stressed the importance of considering ‘not just [the] medical but social and psychological’ factors. The absence of guidance on when advocacy should be initiated compounds this problem.

Participants consistently described IMCA involvement as delaying care. Advocacy exists to support timely, person‐centered decisions, not to impede them. Where involvement adds administrative burden without meaningfully contributing to decision‐making, it works against the interests of the people it is meant to protect. Some services had adapted by initiating the IMCA process alongside waiting‐list placement, a pragmatic but partial solution that does not resolve delayed decision‐making across the range of treatment encountered in SCD.

Although the IMCA is a creature of English and Welsh statute, the dynamics identified here are unlikely to be jurisdictionally confined. Wherever systems provide independent representation for patients without family or friends, whether through advocates, public guardians, or ethics processes, similar questions arise about what triggers involvement, its timing relative to clinical planning, and whether it genuinely shapes decisions or merely endorses them procedurally. The finding that dental treatment was often not regarded as serious, regardless of its extent, may carry particular international relevance given persistent oral health inequalities across many systems [32]. No published research was identified on advocacy in dental decision‐making outside the UK; comparative study of how different legal models perform here would be valuable.

### 5.1 Reflexivity, Strengths and Limitations

The combined clinical and academic backgrounds of the research team inevitably shaped both data generation and analysis [33]. As dentists interviewing dentists, the interviewers (NM, JP, LT) held insider status [33]. Shared professional experience may have  supported rapport and candour about legally sensitive practice, but may also have introduced unexamined shared assumptions and limited probing of the issues that appeared professionally self‐evident. Some participants were recruited through or already known within the researchers' professional networks. This may have eased disclosure but also invited more curated accounts, and the statutory nature of the topic may have prompted socially desirable responses. These influences were addressed rather than eliminated: assumptions were surfaced and debated in discussions before and throughout analysis [20, 34], and JAP, the sole non‐clinical team member, brought an external perspective that questioned interpretations clinicians might otherwise have naturalized. The three interviewers were new to qualitative research; although supported through piloting, senior oversight and concurrent review of transcripts, more experienced interviewers may have probed differently.

The findings represent dentists’ experiences alone. As dentists are responsible for instructing IMCAs, their perspectives warranted primary study; the views of IMCAs, patients, carers, and direct observation of best interests decision‐making remain absent, and are priorities for future research.

Recruitment across a range of service types and locations within England and Wales strengthened the study's ability to capture diverse experiences, though there may have been an element of selection bias as NM, JP, and LT recruited via networks from which they were linked. While the sample's information power was judged sufficient for this specific aim, a larger sample, or one including newly qualified and late‐career dentists, may have generated additional perspectives. Participants were drawn only from jurisdictions where the MCA applies, excluding Scotland and Northern Ireland. The transferability of findings to non‐dental surgical settings may be limited given dentistry's unique contextual features, though parallels exist with other hospital‐based teams managing patients across treatment modalities.

### 5.2 Recommendations

Based on the findings of this study, the following recommendations for clinical practice are proposed:

- Dental services should establish clear, locally agreed criteria for IMCA appointment that move beyond treatment modality alone, incorporating the functional, psychological, and personal impact of both proposed treatment and non‐treatment for the individual concerned.
- Where GA or complex behavior support is being considered for an unbefriended patient assessed to lack requisite capacity, IMCA referral should be initiated prior to definitive treatment plans being produced, rather than to ‘rubber stamp’ a confirmed plan.
- Advocacy services and dental teams should develop shared training or briefing resources to improve IMCAs' understanding of dental procedures, dental behavior support options, and the significance of oral health to quality of life, enabling more meaningful and informed advocacy.

## 6 Conclusion

Dentists in SCD are committed to person‐centered, legally compliant decision‐making for unbefriended patients. However, ambiguity around the dental‐specific meaning of ‘serious medical treatment’ was reported to drive inconsistent IMCA appointment, and advocacy support was described as variable, typically confirming rather than changing the proposed clinical plan and introducing counterproductive delays. Seriousness should reflect not modality alone but the clinical, functional, and personal impact of treatment and non‐treatment. Although the IMCA is specific to England and Wales, comparable safeguards exist worldwide, from public guardians to court‐appointed representatives, and the challenges identified here, (such as unclear thresholds, late engagement, and advocacy that struggles to influence plans), are likely to arise wherever they operate. Cross‐jurisdictional research into how such systems function in dental care is needed to ensure these protections reach those least able to represent themselves.

## Author Contributions

**Andrew Geddis‐Regan**: study conceptualization, ethical process, data generation, data analysis, initial paper write‐up. **Naomi Murray**: data generation, data analysis, drafting and revision of manuscript. **Lucy Taylor**: data generation, data analysis, drafting and revision of manuscript. **Jeena Patel**: data generation, data analysis, drafting and revision of manuscript. **Jordan A. Parsons**: study planning, review of analysis and revision of manuscript.

## Funding

No funding was received for this work.

## Ethics Statement

The authors declare that appropriate ethical approval has been received for the research presented here and that proof of ethical approval can be produced on request. The study was exempt from formal review (UoM reference: 2025‐23392‐42623).

## Conflicts of Interest

The authors declare no conflicts of interest.

## Supporting information




**Supporting File**: scd70276‐sup‐0001‐SuppMat.docx.

## Endnotes

1
*Dental Officers and Senior Dental Officers are clinicians with additional training and experience who may not hold specialist registration with the UK General Dental Council (GDC). They typically work in community dental services, where clinics are equipped with additional facilities and support to manage patients with complex needs. Specialty trainees are dentists undertaking approved postgraduate training leading to GDC specialist registration in Special Care Dentistry. Specialists have achieved GDC specialist registration in Special Care Dentistry, while consultants are the most senior clinicians within this specialty, providing expert clinical leadership alongside specialist practice*.
