# Supplementary material for: Involving Independent Mental Capacity Advocates (IMCAs) in Dental Care Decision‐Making: A Qualitative Exploration of Dentists’ Experiences in England and Wales
Source: Spec Care Dentist. 2026 Oct 22;46(5):e70276. doi: 10.1111/scd.70276 (PMC13688707; doi:10.1111/scd.70276)
Supplement: Supplementary file 1 [file SCD-46-0-s001.docx]

**Involving Independent Mental Capacity Advocates (IMCAs) in Dental Care Decision-Making: an exploration of dentists’ experiences**

**TOPIC GUIDE**

**Ensure consent script is read, and agreement from participant. Once done, check participant is prepared to start recording. Start recording ,then begin interview.**

**Opening question/Ice breaker**

- Can you tell me a little bit about yourself and your current area of work?
- Have you always worked in this setting – explore career history and experience
- What made you chose to work in [setting]
- Can you tell me about your training in this context?

**Moving on to experience of complex decision-making**

- Tell me about your experience with capacity assessments
- How do you handle difficult or uncertain situations with regard to capacity?

**Moving on to IMCA**

- Do you have a policy in your workplace for IMCA/treatment for those lacking capacity?
  - Have they read it, is it up-to-date etc.
- Tell me about your experience with appointing IMCAs: how does this work where you are?
- Who is the IMCA service / provider?
- What situations make you appoint IMCA? Why?
- How helpful or valuable have you found the service?
- Has it ultimately changed treatment provided? For all patients? For some?

IMCA required for ‘Serious Medical Treatment’ in unbefriended people: what does this term mean to you? Is it clear? What do you think it means in the dental context?

**IMCA for LA, GA, Sedation**

- Do you appoint for all **treatment** in unbefriended people? OR just some? WHY? What affects decision to appoint
- Do you use IMCA for all modalities (LA, GA, sedation, non-treatment decisions) or just for some situations? Why?

**IMCA role**

- Have you ever not appointed IMCA and then felt it would have been beneficial?
- How does IMCA appointment impact upon treatment ultimately delivered?
  - Does it change what is offered? Do timescales change?
- Tell me about good experiences of working with IMCA
- What challenges do they face working with IMCA
- Tell me some scenarios or examples where you have or have not appointed and how this has affected next steps?
- How do you feel IMCA role impacts your approach as decision-maker?
- Do you have or follow any guidance or policies in this regard

**DEPENDNIG ON ANSEWRS:**

Example scenarios:

IV sedation: do you appoint IMCA for this or just in some situations?

What if aesthetic work e.g. inhalation sedation for loss of anterior teeth that we cannot replace?

What is simple extraction with LA? WHY would these differ?
